# Identification of Key Proteins from the Alternative Lengthening of Telomeres-Associated Promyelocytic Leukemia Nuclear Bodies Pathway

**DOI:** 10.3390/biology11020185

**Published:** 2022-01-25

**Authors:** Isaac Armendáriz-Castillo, Katherine Hidalgo-Fernández, Andy Pérez-Villa, Jennyfer M. García-Cárdenas, Andrés López-Cortés, Santiago Guerrero

**Affiliations:** 1 Latin American Network for the Implementation and Validation of Clinical Pharmacogenomics Guidelines (RELIVAF-CYTED), 28001 Madrid, Spain; isaac.arcas@gmail.com (I.A.-C.); katherine99.hidalgo@gmail.com (K.H.-F.); andypzvi@gmail.com (A.P.-V.); jennyfergrc7@gmail.com (J.M.G.-C.); 2 Facultade de Ciencias, Universidade da Coruña, 15071 A Coruña, Spain; 3 Instituto Nacional de Investigación en Salud Pública, Quito 170136, Ecuador; 4 Escuela de Medicina, Facultad de Ciencias Médicas de la Salud y de la Vida, Universidad Internacional del Ecuador, Quito 170113, Ecuador

**Keywords:** ALT, PML, telomeres, Pan-Cancer, TCGA, omics

## Abstract

**Simple Summary:**

The alternative lengthening of telomeres is a telomere maintenance mechanism used by some cancer types to elongate their telomeres without the aid of telomerase. This mechanism contributes to the proliferation and immortality of cancer cells. One of the hallmarks of this mechanism is the interaction with promyelocytic leukemia nuclear bodies, which are suspected to be the key places where telomere extension occurs. Despite the discovery of some mechanisms, elements, key genes, and proteins from the pathway, the alternative lengthening of telomeres mechanism is still poorly understood, and it is highly associated with a poor prognosis. In this study, we combined multiomics approaches with genomic, transcriptomic, and proteomic analyses of 71 genes/proteins related to promyelocytic leukemia nuclear bodies in more than 10,000 cancer samples from The Cancer Genome Atlas Consortium. As a result, 13 key proteins were proposed as candidates for future experimental studies that will validate these proteins as therapeutic markers, which will improve the understanding and treatment of these type of cancers.

**Abstract:**

Alternative lengthening of telomeres-associated promyelocytic leukemia nuclear bodies (APBs) are a hallmark of telomere maintenance. In the last few years, APBs have been described as the main place where telomeric extension occurs in ALT-positive cancer cell lines. A different set of proteins have been associated with APBs function, however, the molecular mechanisms behind their assembly, colocalization, and clustering of telomeres, among others, remain unclear. To improve the understanding of APBs in the ALT pathway, we integrated multiomics analyses to evaluate genomic, transcriptomic and proteomic alterations, and functional interactions of 71 APBs-related genes/proteins in 32 Pan-Cancer Atlas studies from The Cancer Genome Atlas Consortium (TCGA). As a result, we identified 13 key proteins which showed distinctive mutations, interactions, and functional enrichment patterns across all the cancer types and proposed this set of proteins as candidates for future ex vivo and in vivo analyses that will validate these proteins to improve the understanding of the ALT pathway, fill the current research gap about APBs function and their role in ALT, and be considered as potential therapeutic targets for the diagnosis and treatment of ALT-positive cancers in the future.

## 1. Introduction

Telomeres are nucleoprotein complexes composed of tandem repeats of TTAGGG, whose primary function is to protect the ends of chromosomes against end-to-end fusions, chromosomal rearrangements, and genomic instability [1,2]. In somatic cells, due to cell division, telomeres shorten, causing senescence or apoptosis [3]. To avoid replicative senescence during tumorigenesis, telomerase reactivates in most types of cancer [3]. However, 10% to 15% of cancers use a telomerase-independent mechanism to preserve their telomeres, called alternative lengthening of telomeres (ALT) [2]. Some pathways and molecular mechanisms of ALT are not yet understood, but it has been proposed that it may use dependent or independent mechanisms of homologous recombination (HR) [4].

A particular hallmark of ALT^+^ cells is the formation of an interactome with promyelocytic leukemia (PML) nuclear bodies, known as ALT-associated PML bodies (APBs) [5,6]. PML bodies are membrane-less organelles found in the cell nucleus, which contain small ubiquitin-like modification (SUMO) sites [7] and are formed by PML, Sp100, and SUMO-1/2/3 proteins. Additionally, they use more than 50 proteins such as RAD52, RAD51, RAD50, RPA, BLM, and BRCA1, among others, which are involved in different cellular functions, such as tumoral suppression, DNA replication, gene transcription, DNA damage response (DDR), senescence, and apoptosis [8,9]. In the course of APBs formation, all of the six subunits (TRF1, TRF2, POT1, TPP1, TIN2, and Rap1) that constitute the shelterin complex detach from the telomeric DNA and are incorporated into the APBs (SUMOylation of shelterin), creating a recombigenic microenvironment that contributes to ALT triggering [8]. 

Normally, the PML bodies are disassembled when cells enter mitosis; however, due to their hyper-SUMOylated state, APBs have been observed in metaphases of cancer cell lines [7]. Recent studies have shown that telomere clustering in tumor cells promotes ALT through mitotic DNA synthesis (MiDAS) [5]. By applying the ATSA (ALT telomere DNA synthesis in APBs) assay, Zhang et al. 2019, demonstrated that telomeric DNA synthesis in ALT^+^ cells takes place exclusively in APBs while in the G2 phase of the cell cycle. In addition, the knockdown of the *PML* gene in ALT^+^ cells resulted in a reduction of telomeres length and decreased ALT function [10]. 

Despite having demonstrated that APBs are essential for the ALT pathway, many of the molecular mechanisms for their assembly and how telomeres cluster inside the PML bodies are still unknown [3,7]. Furthermore, the molecular mechanisms behind the ALT pathway are still poorly understood [10]. In a previous research, we identified a group of 20 genes/proteins that could be used as potential molecular markers for the study of ALT [11].

Within this context, the aim of this study is to evaluate the genomic, transcriptomic and proteomic alterations of 71 genes/proteins associated with APBs by using an integrated TCGA Pan-Cancer Atlas and multi-omics analyses in order to improve the understanding of the role of APBs in cancer, their correlation with ALT, and their application as potential molecular markers for the diagnosis and treatment of ALT^+^ cancers. 

## 2. Materials and Methods

### 2.1. Gene/Protein Set

TelNet (http://www.cancertelsys.org/telnet/ accessed on 2 July 2021) is a database that groups more than 2000 human telomere maintenance (TM) genes. All genes are annotated according to their classification of telomere maintenance mechanism, telomere maintenance function, and a significance score given by the evidence of gene function in telomeres [12]. The database shows the role of each gene in ALT and telomerase-mediated mechanisms. Therefore, the TelNet database was downloaded and manually filtered, resulting in a set of 71 genes that are related to PMLs and APBs (Table S1 in Supplementary File S1). 

### 2.2. TCGA Pan-Cancer Studies Frequencies and OncoPrint of Genomic and Proteomic Alterations

After selecting the set of APBs-related genes/proteins, we analyzed their genomic, transcriptomic, and proteomic alterations in 32 cancer studies from the Pan-Cancer Atlas (PCA) project which is part of The Cancer Genome Atlas (TCGA) consortium [13,14]. With the aid of the cBio Portal database (http://www.cbioportal.org/ accessed on 5 July 2021) [15,16], a total of 10,918 samples were selected from the 32 PCA studies: acute myeloid leukemia (LAML), adrenocortical carcinoma (ACC), bladder urothelial carcinoma (BLCA), brain lower grade glioma (LGG), breast invasive carcinoma (BRCA), cervical squamous cell carcinoma (CESC), cholangiocarcinoma (CHOL), colorectal adenocarcinoma (COAD), diffuse large B-cell lymphoma (DLBC), esophageal adenocarcinoma (ESCA), glioblastoma multiforme (GBM), head and neck squamous cell carcinoma (HNSC), kidney chromophobe (KICH), kidney renal clear cell carcinoma (KIRC), kidney renal papillary cell carcinoma (KIRP), liver hepatocellular carcinoma (LIHC), lung adenocarcinoma (LUAD), lung squamous cell carcinoma (LUSC), mesothelioma (MESO), ovarian serous cystadenocarcinoma (OV), pancreatic adenocarcinoma (PAAD), pheocromocytoma and paraganlioma (PCPG), prostate adenocarcinoma (PRAD), sarcoma (SARC), skin cutaneous melanoma (SKCM), stomach adenocarcinoma (STAD), testicular germ cell tumors (TGCT), thymoma (THYM), thyroid carcinoma (THCA), uterine carcinosarcoma (UCS), uterine corpus endometrial carcinoma (UCEC), uveal melanoma (UVM) [13,14,17,18,19,20,21,22,23,24]. The cBioPortal uses data from the GISTIC2.0 computational approach, which facilitates sensitive and confident localization of CNV (copy number variation) amplifications and deep deletions in human cancers [25]; additionally, it identifies in-frame, truncating, and missense mutations through whole-exome sequencing; mRNA up and downregulation are analyzed through RNA sequencing V2 RSEM by comparing the expression Z-scores of tumor samples to the logarithmic expression of mRNA of adjacent normal samples [26] and the up and downexpression of proteins are measured by reverse-phase protein arrays (RPPA) [27]. 

To calculate the frequency means of every genomic, transcriptomic, and proteomic alteration and construct the OncoPrint we: (1) filtered and calculated the number of alterations per gene and per PCA type; (2) calculated the frequency of alteration of each gene through normalization by dividing the number of alterations by the number of individuals of each cancer study; (3) identified the most altered APBs genes/proteins with the aid of a boxplot by using Tukey’s test; (4) validated the most significantly altered genes/proteins with a multiple comparison test by using the original false discovery rate (FDR) method of Benjamini and Hochberg using GraphPad Prism v9.1.1 software (*p* < 0.01) [28]

### 2.3. Protein–Protein Interaction Network

In order to predict the most essential protein interactions, an APBs protein–protein interaction (PPi) network was constructed with the aid of the STRING database (https://string-db.org/ accessed on 10 August 2021). An interaction score of 0.9 (highest confidence) was set according to coexpression, curated from the database, and experimentally determined [29,30]. The most significant signaling pathways (*p* < 0.001) related to APBs were selected and classified in the network. 

### 2.4. Functional Enrichment Analysis

An enrichment analysis gives curated signatures of protein sets generated from omics experiments [31]. Thus, we performed the analysis of the 71 APBs proteins by using the g:Profiler tool version e104_eg51_p15_3922dba (https://biit.cs.ut.ee/gprofiler/gost accessed on 12 August 2021) [32]. The most significant annotations were selected after Benjamini–Hochberg and false discovery rate (FDR) corrections (*p* < 0.001), based on gene ontology (GO), molecular function (MF), biological process (BP), the Kyoto Encyclopedia of Genes and Genomes (KEGG) and REACTOME signaling pathways [32,33]. 

### 2.5. Correlation between ALT and APBs in the Pan-Cancer Studies

In our former study [11], ALT tumors were classified as frequent ALT tumors, rare ALT tumors, and non-ALT tumors. For this study, we wanted to determine whether the key APBs proteins identified in this study have a correlation with the ALT proteins identified in our latter study. Under this context we: (1) elaborated a Venn diagram to correlate the type of ALT tumor with the PCA studies with the most significant patterns of genomic, transcriptomic, and proteomic alterations; (2) constructed protein–protein interaction (PPi) networks to predict the interactions among the APBs proteins with the highest frequencies of alterations and the most altered proteins from each ALT-frequent and ALT-rare groups determined in our previous study; and (3) with the aid of a Venn diagram we integrated four different approaches in order to predict key proteins from the APBs pathway. 

## 3. Results

### 3.1. Gene Set and Genomic, Transcriptomic, and Proteomic Alterations

To evaluate the genomic, transcriptomic, and proteomic alterations, we selected 71 APBs-related genes/proteins (Table S1); these were analyzed in the cBioPortal [15,16] by selecting 10,918 samples from 32 studies of the Pan-Cancer Atlas (PCA) [13,14,17,18,19,20,21,22,23,24] from The Cancer Genome Atlas consortium (TCGA).

**Figure 1:**
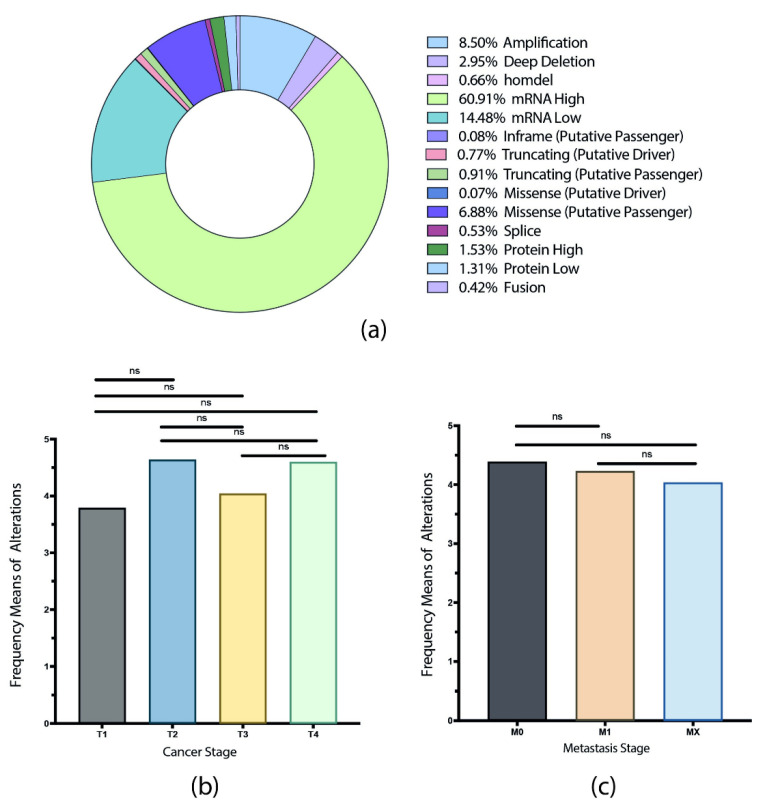
Genomic, transcriptomic, and proteomic alterations. (**a**) A donut chart with the most frequent alterations identified in the 71 APBs genes/proteins analyzed. (**b**,**c**) Alterations observed in each cancer and metastasis stage of the PCA studies showing no significant difference among alterations in each stage. ns: non-significant.

A total of 72,492 alterations were identified and a donut chart was elaborated showing the most frequent alterations after all values were normalized by the number of samples in each study (Table S2 in Supplementary File S2). Figure 1a, shows that the most frequent genomic alteration was mRNA high (61%), followed by mRNA low (14.5%), copy number variation (CNV) amplifications (8.50%), missense (putative passenger) mutations (6.90%), deep deletions (2.95%), and protein high and low with 1.53% and 1.31%, respectively.

To understand the implication of APBs genes/proteins alterations in cancer progression from primary tumors to metastasis (T1 to T4), genomic, transcriptomic, and proteomic alterations were subgrouped for each cancer and metastasis stage from the PCA studies, when available. PCA studies with tumor stage data were (n = 7406): ACC, BLCA, BRCA, CESC, CHOL, COAD, ESCA, HNSC, KICH, KIRC, KIRP, LIHC, LUAD, LUSC, MESO, PAAD, SKCAM, STAD, TGCT, and THCA; while PCA studies with metastasis stage data were (n = 6697): BLCA, BRCA, CESC, CHOL, COAD, ESCA, HNSC, KICH, KIRC, KIRP, LIHC, LUAD, LUSC, MESO, PAAD, SKCM, STAD, TGCT, and THCA. All alterations were normalized by the number of samples in each stage for each study. No significant differences were found between cancer stage alterations (Figure 1b) or metastasis stage alterations (Figure 1c) after a multiple comparison with the original false discovery rate (FDR) method of Benjamini and Hochberg (*p* < 0.001).

### 3.2. TCGA Pan-Cancer Studies Frequencies and OncoPrint of Genomic Transcriptomic and Proteomic Alterations

**Figure 2:**
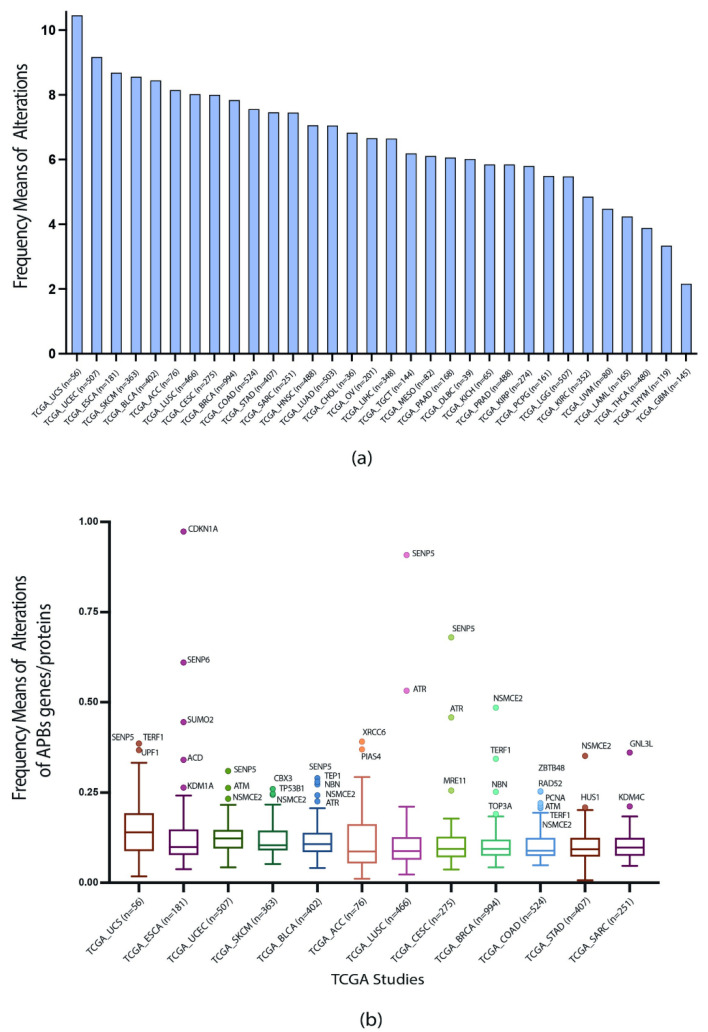
Frequency of genomic, transcriptomic, and proteomic alterations per TCGA Pan-Cancer type. (**a**) Frequency means of alterations of the 71 APBs genes/proteins across the 32 PCA studies. (**b**) Boxplot showing genes/proteins (data points), which showed significant differences in alterations patterns in twelve PCA studies (x axis).

With the values of genomic, transcriptomic, and proteomic alterations normalized by the number of samples in each study, the highest frequency means of alterations were calculated for the 32 studies and 71 genes/proteins (Table S3 in Supplementary File S3). UCS was the cancer type with the highest alteration frequency mean (10.456) and GBM with the lowest alteration frequency mean (2.157) (Figure 2a).

Consequently, to identify highly altered genes/proteins, the first quartile of the PCA studies with the highest means of alterations were selected to construct a boxplot, and genes and proteins that showed significantly different patterns of alterations were identified by using Tukey’s test. Figure 2b shows twelve types of PCA studies in which the APBs genes/proteins present the highest number of frequency means alterations (Table S4 in Supplementary File S4). SENP5, 2TRF1, UPF1, NSMCE2, CDKN1A, SUMO2, ACD, KDM1A, ATM, CBX3, TP53B1, TEP1, NBN, ATR, PIAS4, XRCC6, MRE11, TOP3A, SBTB48, RAD52, HUS1, and GNL3L genes/proteins showed significantly higher means of genomic, transcriptomic, and proteomic alterations across the twelve studies, therefore, they can be considered as targets of interest for the following analyses.

**Figure 3:**
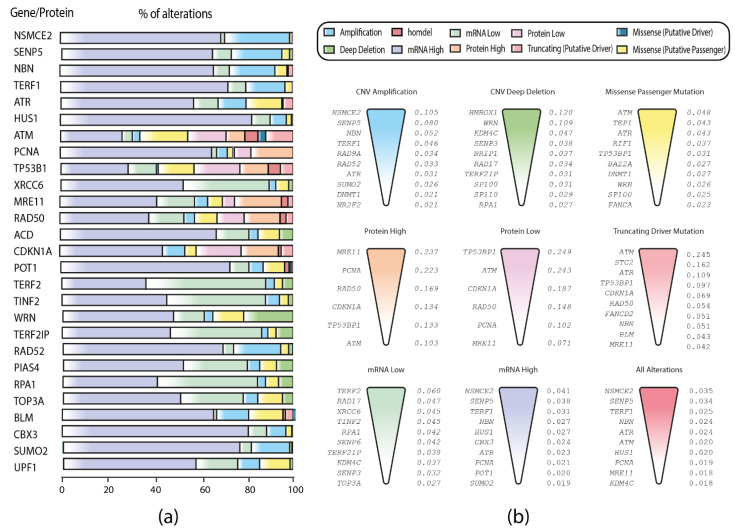
OncoPrint and ranking of the genes/proteins with the highest means of alterations across the PCA studies. (**a**) OncoPrint of genomics, transcriptomics, and proteomic alterations across 32 TCGA Pan-Cancer studies. (**b**) Ranking of the most altered genes/proteins per alteration type.

Furthermore, an OncoPrint with the first two quartiles of the genes/proteins with the highest means of genomic, transcriptomic, and proteomic alterations was constructed by using the cBioPortal data (https://www.cbioportal.org/ accessed on 5 July 2021) [15,16] (Figure 3a). The most common alteration type observed was mRNA high, followed by mRNA low and CNV amplification (Table S5 in Supplementary File S5). In addition, genes/proteins with the highest alteration frequencies were NSMCE2, SENP5, and TRF1 with mRNA high alterations; TRF2, RAD17, and XRCC6 with mRNA low; NSMCE2, SENP5, and NBN with CNV amplification; HMBOX1, WRN, and KDM4C with CNV deep deletion; ATM, TEP1, and ATR with missense mutations; ATM, STC2, and ATR with truncating mutations; MRE11, PCNA, and RAD50 with protein high; TP53B1, ATM, and CDKN1A with protein low; NSMCE2, SENP5, and TRF1 in the overall alterations (Figure 3b). 

**Figure 4:**
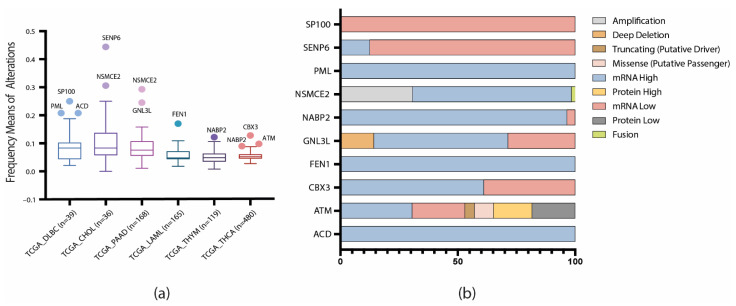
Frequency and patterns of genomic, transcriptomic, and proteomic alterations (data points) in non-ALT tumors (x-axis). (**a**) Boxplot showing 10 genes/proteins with significant differences in alteration frequencies in non-ALT tumors. (**b**) OncoPrint showing the main alterations of the 10 APBs-related genes/proteins in non-ALT tumors.

Finally, in order to analyze how the APBs-related genes/proteins behave in tumors not associated with ALT, we constructed another boxplot with the following PCA studies: DLBC, CHOL, PAAD, LAML, THYM, and THCA (Figure 4a). As a result, the following genes/proteins showed abnormal patterns of mutations: ACD, ATM, CBX3, FEN1, GNL3L, NABP2, NSMCE2, PML, SENP6, and SP100. As a consequence, an OncoPrint showing the genomic, transcriptomic, and proteomic alterations of these genes/proteins was constructed. Figure 4b, shows that mRNA up and downregulation are the dominant mutations in the non-ALT tumors; the implication of these alterations in these genes/proteins is discussed later. 

### 3.3. Protein–Protein Interaction (PPi) Network and Functional Enrichment Analysis

**Figure 5:**
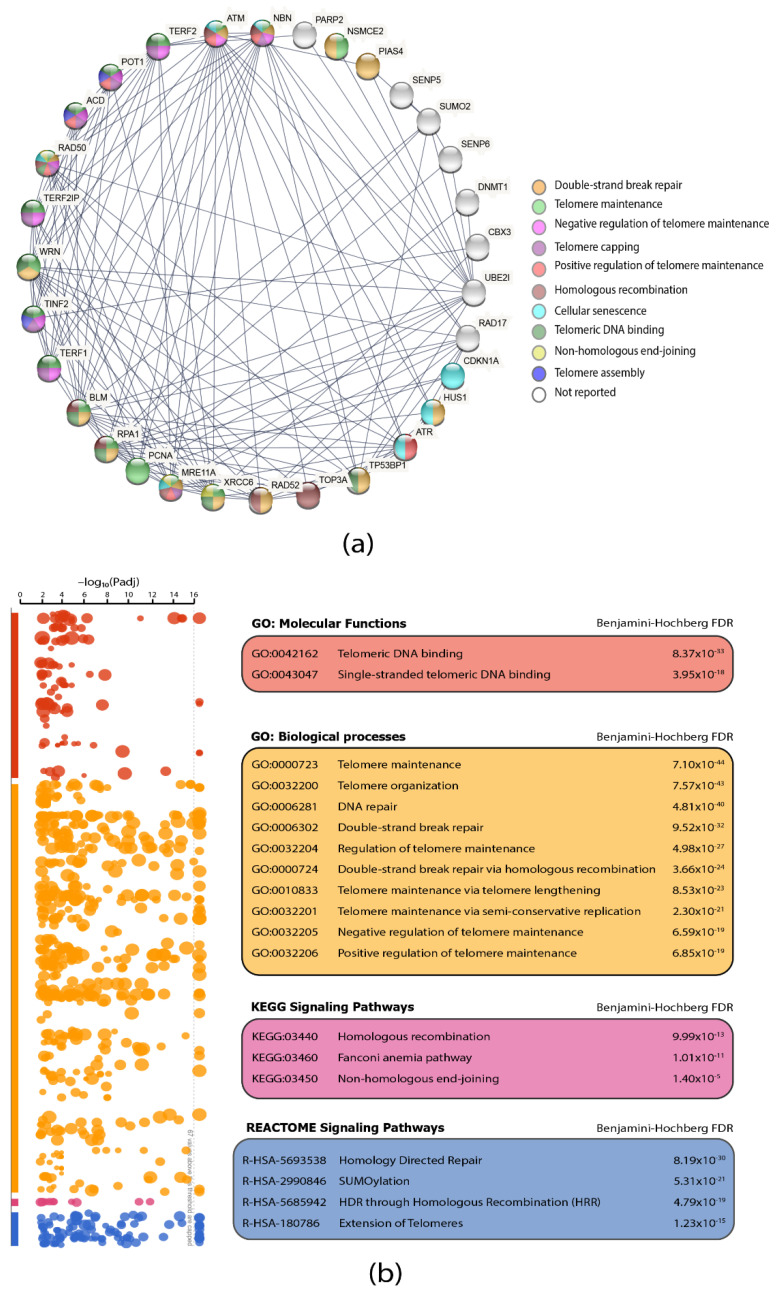
Protein–protein interaction network and functional enrichment analysis. (**a**) Protein–protein interaction network showing APBs proteins interactions. Proteins are colored according to the most significant pathway they are involved in. (**b**) Functional enrichment analysis showing the most significant GO: biological processes, GO: molecular functions, and KEGG and REACTOME signaling pathways according to Benjamini–Hochberg FDR (*p* < 0.001); data were visualized using the g:profiler software.

PPi networks are fundamental resources to understand protein interactions among diseases [34]. Thus, we analyzed the 71 APBs-related proteins selected for our study by querying the STRING database [29]. After selecting the interaction score of the highest confidence (0.900) [35], according to the level of evidence of interactions, we obtained a network with 31 proteins interacting at the highest level of evidence, of which 23 are involved in pathways significantly related (*p* < 0.001) to the molecular functions linked to the mechanism of formation and function of APBs. Figure 5a shows interactions between proteins which are differentiated by colored nodes according to the most significant pathway in which each one is intervening; 70% of these are involved in telomeric DNA binding, 56% are involved in double-strand break repair (DSBR) and in telomere maintenance, 40% in telomere capping and positive and negative regulation of telomere maintenance, and around 30% in homologous recombination and non-homologous end-joining (NHEJ) mechanisms.

Additionally, a functional enrichment analysis of the 71 proteins was performed using the g:profiler software [32] (Table S6 in Supplementary File S6). Figure 5b shows a Manhattan plot of the most significant GO: molecular functions, GO: biological processes and KEGG and REACTOME [36] signaling pathways with Benjamini–Hochberg FDR (*p* < 0.001), which gives us a clearer idea about the function of the studied proteins and allows us to understand and discuss the consequences of their genomic alterations in the different types of cancer.

### 3.4. Correlation between ALT and APBs in TCGA PCA Studies

**Figure 6:**
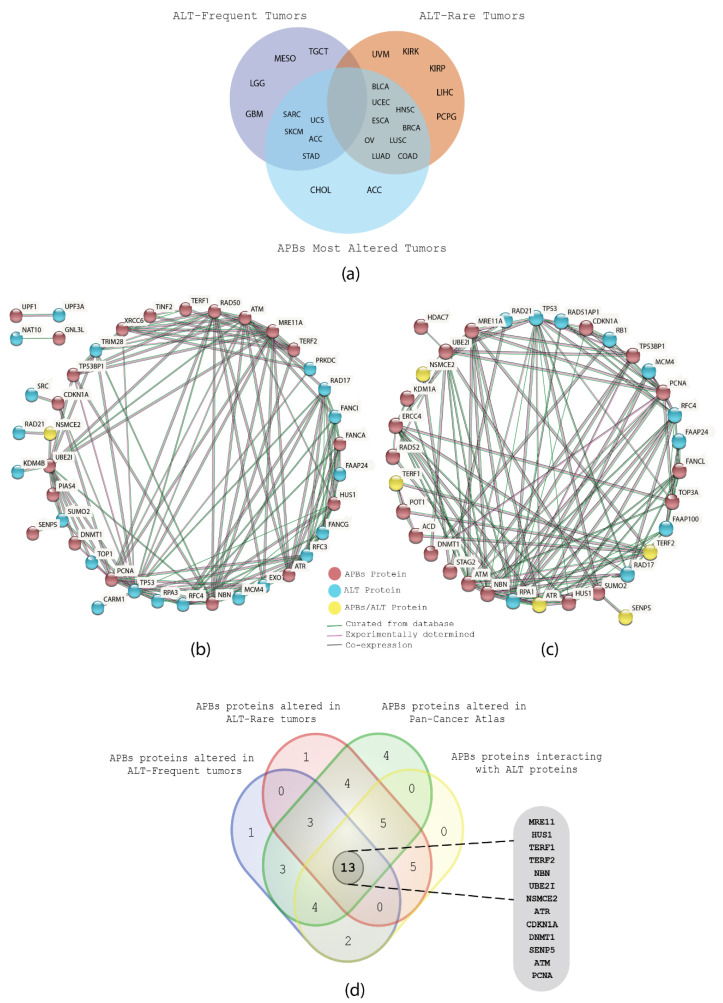
Correlation of APBs-related and ALT-related proteins. (**a**) Venn diagram showing the association of ALT-frequent and -related tumors with the PCA tumors with the highest means of alterations in the APBs proteins. (**b**) PPi network showing the interaction among ALT-frequent tumors proteins and APBs proteins. (**c**) PPi network showing the interaction among ALT-rare tumors proteins and APBs proteins. (**d**) Venn diagram showing an integrative analysis of different in silico approaches that resulted in the obtention of the 13 most relevant proteins of the study.

In our previous study [11], we classified the PCA tumors based on literature reports and in silico analyses as: frequent, rare, and non-ALT tumors. With the help of a Venn diagram (Figure 6a), a correlation was made among the ALT-related tumors from our previous study and the most altered tumors across the 32 PCA types of the present study with APBs. Consequently, it was observed that SARC, SKCM, UCS, ACC, and STAD (ALT frequent tumors) and BLCA, UCEC, ESCA, COAD, LUSC, and BRCA (ALT rare tumors) show a high frequency of genomic, transcriptomic, and proteomic alterations of APBs-related genes/proteins. 

As a result, the most altered proteins from the ALT frequent and rare tumors mentioned above were selected and a protein–protein interaction was performed with the most altered APBs proteins from the same PCA studies by using the same criteria of evidence and interactions used in the construction of the previous network. Figure 6b shows the interactions between the APBs-related and ALT-related proteins from the frequent ALT tumors, and Figure 6c shows the interactions between the same groups of proteins but from the rare ALT tumors. Thus, the interaction of ALT and APBs proteins and their genomic, transcriptomic, and proteomic alterations can be correlated to improve the understanding of their association in the activation of telomerase-independent telomere maintenance mechanisms in cancer. 

Then, to prioritize and identify a set of key proteins from the APBs, we integrated the most significant proteins from the networks in Figure 5a and Figure 6b,c and the OncoPrint analysis in Figure 3a,b. As a result, Figure 6d shows a Venn diagram with the integrated analysis of the most significant APBs proteins from the different in silico approaches applied, resulting in 13 key proteins. 

**Figure 7:**
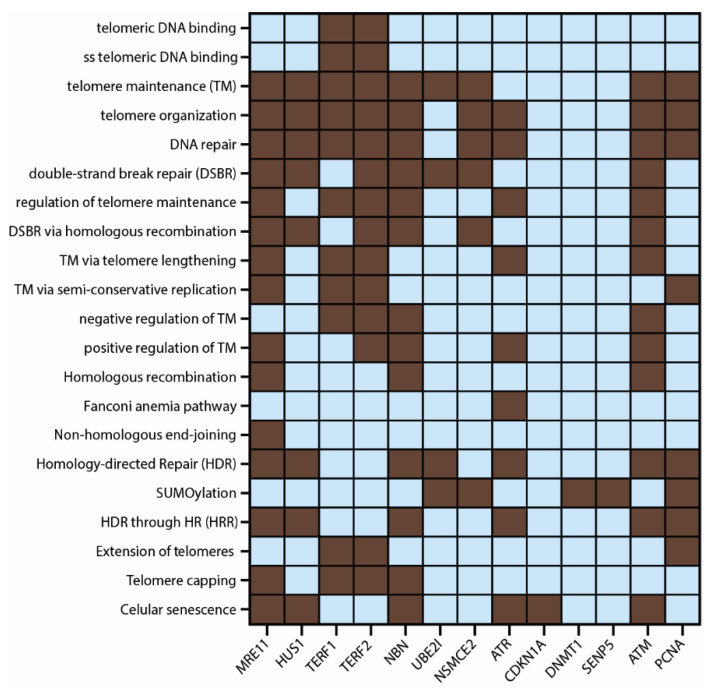
Heatmap showing the 21 most significant GO processes, functions, and signaling pathways (dark boxes) in which the 13 most relevant APBs proteins obtained in this study are involved.

Finally, Figure 7 shows a heatmap with the most significant (*p* < 0.001) GO processes, functions, and signaling pathways where these 13 APBs proteins are interacting according to the protein–protein interaction network and functional enrichment analysis performed in previous steps. A total of 21 pathways related to TM mechanisms were selected and all of them are related to telomere maintenance mechanisms. 

## 4. Discussion

The alternative lengthening of telomeres mechanism is a break-induced replication (BIR)-based process, through which some cancer cells elongate their telomeres without the need of telomerase [37]. Although ALT has been widely studied and described in recent years, the mechanism through which it is activated and most of its pathways are still poorly understood [6]. One of the hallmarks of ALT is its association with promyelocytic leukemia nuclear bodies, better known as APBs [38]. APBs formation is driven by liquid–liquid phase separation with an environment marked by high levels of SUMOylated proteins, that bring telomeres together allowing ALT to occur; however, the way APBs assemble or how they promote ALT remains unclear [39]. As a consequence, for this study we used a multiomics approach to identify the genomic, transcriptomic, and proteomic mutations of 71 APBs-related genes in 32 cancer types from TCGA Pan-Cancer Atlas; as a result, we proposed 13 key proteins, which, in addition to the 20 ALT-related proteins proposed in a past study [11], represent the best in silico evidence so far for the study of the ALT mechanism in cancer. 

A total of 72,492 alterations were identified (Figure 1), the mRNA alterations and copy number variation (CNV) amplifications being the predominant mutations. mRNA up and downregulation is a measure to quantify the expression level of a certain gene and how it will correlate or affect protein expression as a post-transcriptional level, while CNV describes how the number of copies of a gene can be higher or lower from one individual to another, which can result in a loss or gain of functions [40]; in cancer, there is a close correlation between CNV and differential gene expression at a transcriptional level, therefore, the correlation of these alterations in the APBs-related genes may help to explain its function in the ALT pathway. ALT is known to commonly occur in only 10% to 15% of cancers, most of them from mesenchymal origin [41]; however, in recent years, ALT^+^ cells have been observed in a wide variety of epithelial tumors, and there is strong evidence of switching from a telomerase-mediated telomeric extension to ALT, as a consequence of anti-telomerase and radiation-based therapies, which can trigger the accumulation of DNA damage response (DDR) factors in telomeres that can lead to ALT activation [42,43]. 

With the first quartile of PCA studies exhibiting the highest frequencies of alterations, we constructed a boxplot applying the Tukey´s test and as a result, the genes/proteins with different patterns of genomic, proteomic, and transcriptomic alterations were determined. Figure 2b showed genes/proteins SENP5, TRF1, UPF1, NSMCE2, CDKN1A, SUMO2, ACD, KDM1A, ATM, CBX3, TP53B1, TEP1, NBN, ATR, PIAS4, XRCC6, MRE11, TOP3A, SBTB48, RAD52, HUS1, and GNL3L to have the highest frequencies of alterations among the different PCA studies. Then, we wanted to observe the predominant mutations of each gene/protein, therefore, an OncoPrint with the first quartile of genes/proteins with the highest frequencies of alteration was constructed with the aid of alterations data from the cBioPortal [15,16]; Figure 3a showed the predominant alterations of each gene/protein, where mRNA high had the highest percentage of alterations; nevertheless, XRCC6, TRF2, ATM, TP53B1, MRE11, RAD50, CDKN1A, TINF2, WRN, and RPA1 are genes/proteins with a different pattern of alterations, hence, we decided to rank each gene/protein per alteration; Figure 3b showed each genomic, transcriptomic or proteomic alteration with a ranking of the most altered gene/protein for each one. This ranking allowed us to elucidate the role of each gene/protein in APBs. For instance, NSMCE2 was highly amplified and overexpressed across the PCA studies (Figure 3b); this gene encodes a protein of the small ubiquitin-related modifier (SUMO) and it is part of the SMC5/6 complex, which is crucial for the SUMOylation of proteins [44] that is a hallmark of the APBs environment. Additionally, the knockdown of NSMCE2 in ALT^+^ cell lines had led to a reduction of telomere length [45]. 

In order to observe how the dataset behaves in tumors not associated with ALT, we constructed a boxplot with these PCA studies and observed 10 genes/proteins with abnormal alterations frequencies in these cancers (Figure 4a). Furthermore, with the aid of an OncoPrint (Figure 4b), we identified mRNA up and downregulation to be the predominant alterations. SP100, PML, FEN1, and CBX3 have been identified as ALT enhancers [46,47,48]; SP100 is known to induce APBs formation [46] and is downregulated in non-ALT tumors (Figure 4b) while PML, FEN1 and CBX3 are overexpressed. SENP6 and GNL3L are ALT repressors [49,50], the latter is known to bind to TRF1 preventing PMLs formation [50] and is commonly upregulated in non-ALT tumors (Figure 4b). NABP2 and ACD association with ALT is ambiguous [46,51]; ACD, also known as TPP1 is a main component of the shelterin complex, which is important to maintain telomere stability in normal cells; it has been reported that ACD repression promotes ALT in cancer cells [51]; Figure 4b showed ACD to be highly overexpressed in non-ALT tumors. ATM and NSMCE2 showed abnormal alteration frequencies in ALT tumors (Figure 2b); both genes are known enhancers of the ALT pathway and displayed similar alteration frequencies in the non-ALT tumors; therefore, these genes should be considered for future in vivo studies as potential targets in the study of the switching from a telomerase-mediated telomere maintenance mechanism to ALT.

To further understand the role of the proteins analyzed in this study, we constructed a protein interactome among the 71 APBs-related proteins by using the highest confidence score of 0.9 according to coexpression, curated from the STRING database, and experimentally determined parameters. As a result, in Figure 5a, a protein–protein interaction (PPi) network with 31 proteins was observed; the most significant (*p* < 0.001) pathways were DNA double-strand breaks (DDSB), telomere maintenance (TM), negative and positive regulation of telomere maintenance, non-homologous end joining (NHEJ) and homologous recombination (HR). ALT is a BIR-related process, triggered by oxidative stress due to cancer treatment; all NHEJ and HR are BIR-related pathways that are proposed as the main way by which ALT^+^ cells extend their telomeres [52]. In fact, BIR-induced replication stress with the SUMOylation of key proteins initiates the recruitment of DNA damage response (DDR) factors in APBs of ALT^+^ cell lines [53]. Moreover, a functional enrichment analysis showed that Fanconi anemia pathway proteins like FANCD2, which is correlated with APBs formation [47], was highly enriched in our protein set; one report suggests that FANCD2 depletion can lead to a high telomeric extension in APBs and positively regulate H2AX and TP53B1 proteins in ALT cells [53]; Figure 3b showed FANCD2 truncation, which is favorable for ALT activation. 

In our last study, we classified the PCA studies according to literature reports and alteration frequencies as ALT-frequent tumors, ALT-rare tumors, and not reported [11]. With the aim to observe in which category the PCA studies with the highest frequencies of genomic, transcriptomic, and proteomic alterations of this study are, we grouped them in a Venn diagram which can be observed in Figure 6a, showing that SARC, SKCM, UCS, ACC, and STAD are ALT-frequent tumors and BLCA, UCEC, ESCA, OV, HNSC, BRCA, LUSC, LUAD, and COAD are ALT-rare tumors. Then, we constructed two protein interactomes applying the same criteria used for the previous protein–protein interaction network; Figure 6b showed an interactome of ALT-related proteins and APBs-related proteins that have high alteration frequencies in ALT-frequent tumors, while Figure 6c showed the interactions of ALT-related proteins and APBs-related proteins with the highest alteration frequencies in ALT-rare tumors. These interactomes can improve the understanding of how PMLs associate with ALT^+^ cell lines for the assembling of APBs; for instance, HDAC7 is believed to promote PML protein SUMOylation [4], however, how the HDAC family interacts with PMLs is still unclear. In the interactome of Figure 6c, HDAC7 is interacting directly with UBE2I, which is a highly expressed protein in ALT^+^ cancers such as osteosarcoma [54,55]. 

Moreover, we integrated all the multiomics approaches used in the study: PCA genomic, transcriptomic and proteomic alterations, protein interactomes, enrichment functional analysis, and gene ontology in a single Venn diagram (Figure 6d) and identified 13 proteins that have significative alterations and interactions: MRE11 and NBN, which are part of the MRN complex (MRE11/RAD50/NBN), predicted to be a key step in APBs formation [56]; TRF1 and TRF2, components of the shelterin complex, believed to be recruited by APBs with the aid of the MRN complex and PML protein and predicted to be SUMOylated by NSMCE2 in ALT^+^ cell lines [57,58,59]. Furthermore, TRF1 inhibition has been associated with the disassembly of APBs and the TRF1 fusion with the FokI nuclease, which have been observed to induce DSBs that can enhance APBS formation through HDR mechanisms [60,61,62,63]. However, the mechanism behind TRF1/2 interaction with the PML protein in the assembling of APBs remains unclear [58]. CDKN1A protein knockdown is associated with activation of ALT [64]; Figure 3b showed CDKN1A to have lower protein expression in about 20% of the PCA studies and truncating mutations in 10% of cancers. HUS1 is part of the 911 (RAD9-RAD1-HUS1) checkpoint that activates ATR and protects telomere integrity during DNA damage response and oxidative stress [47,65]. SENP5 is a key protein in the SUMOylation pathway [54,66], UBE2I is a positive regulator of APBs [54,55], DNMT1 depletion is related to ALT activation [47] and ATM and PCNA have been observed to colocalize with APBs during HR and HDR [67,68]. Finally, to summarize the role of the 13 key APBs-related proteins, a heatmap was elaborated showing the 21 most significant pathways in which they are interacting (Figure 7). 

## 5. Conclusions

This work identified 13 key APBs-related proteins, which, after a series of integrated in silico and multiomics analyses, showed distinctive genomic, transcriptomic, and proteomic alterations, significant protein–protein interaction patterns and appeared to be involved in significant pathways related to telomere maintenance through APBs. This protein set, in addition to 20 ALT-related proteins identified in a previous study [11], represents, so far, the most complete in silico evidence of potential molecular targets for the study of the ALT pathway. Although this statistically supported prioritization is based on more than 10,000 tumor samples, further ex and in vivo experiments are needed to validate our findings and fill the knowledge gap that currently exists in the ALT pathway molecular research. This should be considered as a limitation of our study. Bioinformatics techniques through the aid of computational biology models have proven to be valuable tools to prioritize proteins that could improve future in vivo research in different hallmarks of cancer progression such as the APBs-mediated telomere maintenance. 

## Data Availability

All data generated for this study are included in the manuscript and its supplementary files.

## Supplementary Materials

The following are available online at https://www.mdpi.com/article/10.3390/biology11020185/s1, Table S1: Gene/protein set. Table S2: Genomic, transcriptomic, and proteomic alterations across the 32 PCA studies. Table S3: TCGA study vs. alteration (normalized). Table S4: TCGA study vs. gene (normalized). Table S5: List of genes from the highest alteration frequency to the lowest according to each mutation type. Table S6: Functional enrichment analysis.
